# Influence of *FKBP5* Variants and Childhood Trauma on Brain Volume in Non-clinical Individuals

**DOI:** 10.3389/fnbeh.2021.663052

**Published:** 2021-06-03

**Authors:** Aeran Kwon, Sungkean Kim, Hyeonjin Jeon, Hyun Seo Lee, Seung-Hwan Lee

**Affiliations:** ^1^Department of Social Welfare and Counseling, Chodang University, Muan, South Korea; ^2^Department of Human-Computer Interaction, Hanyang University, Ansan, South Korea; ^3^Clinical Emotion and Cognition Research Laboratory, Inje University, Goyang, South Korea; ^4^Department of Psychiatry, Inje University, Ilsan-Paik Hospital, Goyang, South Korea

**Keywords:** *FKBP5*, childhood trauma, orbitofrontal cortex (OFC), middle temporal gyrus (MTG), neuroplastic compensatory mechanism

## Abstract

The present study aimed to investigate the possible influence of childhood trauma and its interaction effect with 10 single-nucleotide polymorphisms (SNPs) of the FK506-binding protein 51 (*FKBP5*) gene on brain volume in non-clinical individuals. One hundred forty-four non-clinical volunteers (44 men and 100 women) were genotyped with respect to 10 variants (rs9296158, rs3800373, rs1360780, rs9470080, rs4713916, rs4713919, rs6902321, rs56311918, rs3798345, and rs9380528) of *FKBP5*. Participants underwent magnetic resonance imaging (MRI) scan and psychological assessments such as the childhood Trauma Questionnaire (CTQ), Hospital Anxiety and Depression Scale, rumination response scale, and quality of life assessment instrument. Individuals with the high CTQ score showed enlarged volume of the left orbitofrontal cortex (OFC) if they have childhood trauma-susceptible genotype of *FKBP5* rs3800373, rs1360780, rs4713916, rs4713919, rs6902321, and rs3798345 and enlarged volume of the left middle temporal gyrus (MTG) if they have childhood trauma-susceptible genotype of *FKBP5* rs3800373, rs1360780, rs4713916, and rs3798345. Among those with the childhood trauma-susceptible genotype, the left OFC and left MTG showed significant negative correlations with positive feelings about life, and the left OFC showed significant positive correlations with negative cognition. This is one of the few studies to identify the volume alteration of the left OFC and the left MTG for the *FKBP5* gene–childhood trauma interaction in non-clinical individuals.

## Introduction

The neurobiological mechanisms, where genes and psychosocial environments dynamically interact with each other, influence subsequent brain functions, structures, and psychopathologies (Hyde et al., 2011; Hyde, 2015; Kim et al., 2018). The presence of both genetic variants with risky allele and childhood adversity may synergistically increase the risk of psychopathology (Kim et al., 2018). It has been also reported that individuals with both genetic variants with risk allele and childhood adversity were associated with volume alteration in several regions (Carballedo et al., 2013; Harrisberger et al., 2015). In contrast, those risks have not been observed among individuals with the same gene allele and exposure to childhood adversity (Marusak et al., 2016). Such a difference may be participants’ ethnicity and population differences (González-Castro et al., 2017). However, the mixed results related to the genotype effect were revealed in the same ethnicity (Jin et al., 2019).

Exposure to emotional and physical adversity during childhood has been associated with poor mental and physical health, academic performance, and socio-occupational functioning (McCrory et al., 2011). Childhood trauma could lead to neuroanatomical changes in highly vulnerable regions of the brain (McCrory et al., 2011), which include the amygdala, hippocampus, prefrontal cortex (PFC), visual and auditory cortices, and thalamus (Teicher et al., 2016). Furthermore, studies have identified the effects of childhood adversity on neural correlates of emotional, reward, and cognitive processing, which are commonly associated with the anterior cingulate cortex (ACC) and orbitofrontal cortex (OFC; Hart and Rubia, 2012). According to a recent meta-analysis, individuals exposed to childhood trauma showed distinctive differences in brain volumes when compared to controls: smaller superior temporal gyrus, insula, parahippocampal gyrus and middle temporal gyrus (MTG), and enlarged right superior frontal and left middle occipital gyri (Lim et al., 2014).

Previous studies have reported the relationship between genetic variants and greater susceptibility to psychopathological development in individuals with childhood trauma (Caspi et al., 2002; Binder et al., 2008; Bradley et al., 2008; Weder et al., 2009; Hornung and Heim, 2014). According to the genetic susceptibility model, genetic variations reflect differential sensitivity to environmental factors through the stress response system accompanied by the hypothalamic–pituitary–adrenal (HPA) axis (de Castro-Catala et al., 2017). Stress-response endophenotypes indicate higher dexamethasone suppression test levels or higher odds of psychiatric diagnosis. For their connections to cortisol-induced responses, they have been associated with several neuronal functions and brain structures regarding mood regulation, particularly in the context of childhood trauma (Gerritsen et al., 2017). The FK506-binding protein 51 (*FKBP5*), which is involved in transcriptional regulation of the HPA axis, is one of the major candidate genes (White et al., 2012). *FKBP5* protein of the *FKBP5* gene, which is located on chromosome 6p21 (Binder, 2009), is involved in regulating glucocorticoid receptor activity and providing a negative feedback loop in the HPA axis (Han et al., 2017). The overexpression of this protein can reduce the hormone-binding affinity and nuclear translocation of the glucocorticoid receptor, downregulating the expression of anti-inflammatory proteins in neuronal nuclei (Wochnik et al., 2005). Several studies pointed out that the *FKBP5* gene, with impact of childhood trauma, can predict brain structural alterations (Grabe et al., 2010; White et al., 2012; Fani et al., 2013; Pagliaccio et al., 2014; Holz et al., 2015).

Although the studies to date are difficult to compare, we have identified the inconsistency in previous studies. Some studies have narrowed their focus solely on direct *FKBP5* genetic effects on brain alteration, without factoring in an individual’s life stress (Zobel et al., 2010; Fani et al., 2013; Fujii et al., 2014). Other studies have looked at both the main and interaction effects of *FKBP5* but considered only one or a few single-nucleotide polymorphisms (SNPs) of *FKBP5* (Holz et al., 2015; Grabe et al., 2016; Tozzi et al., 2016). Since they have used a few SNPs in their study, effects of other SNPs in *FKBP5* and childhood trauma exposure on structural alteration in brain could not be fully identified. Furthermore, studies have used discrete variables in childhood trauma exposure (Grabe et al., 2016), which may fail to capture the original characteristics of the continuous scale. Also, to include the total score of childhood trauma scale to measure the effect of childhood trauma experiences seems a more comprehensive approach.

To overcome these inconsistencies, the present study explored multiple *FKBP5* SNPs and their interaction with the environment on brain volume alteration. We included a total of 10 *FKBP5* SNPs (rs9296158, rs3800373, rs1360780, rs9470080, rs4713916, rs4713919, rs6902321, rs56311918, rs3798345, and rs9380528) to investigate their possible interaction effect with childhood trauma. These 10 SNPs have been located in intron 7, Bin3 (site 6) which were differentially methylated in response to childhood trauma in the presence of the *FKBP5* risk allele (Klengel et al., 2013; Yehuda et al., 2016). Some SNPs have been reported as relevant predictors of stress susceptibility and risk factors of psychiatric symptoms (Binder et al., 2008; White et al., 2012; Zannas et al., 2015). Furthermore, the continuous scale was used to assess effects of total childhood trauma.

Given the previous findings, examining SNPs related to methylation at intron 7 of *FKBP5* in the effects of childhood trauma is a cogent approach. The *FKBP5* risk allele carrier and early trauma exposure lead to demethylation of intron 7 CpGs in *FKBP5*, which further amplifies genotype-dependent differences (Klengel et al., 2013). Genetic differences lead to divergent chromatin conformations and interactions of long-range enhancers with the transcription start site. It is related to a differential transcriptional activation of *FKBP5* by glucocorticoid receptor activation depending on childhood trauma. These changes in chromatin structure increased cortisol levels and thus glucocorticoid receptor binding, leading to changes in DNA methylation in intron 7, further increasing the differential responsiveness of *FKBP5* to glucocorticoid receptor activation (Klengel et al., 2013). Holocaust survivors and their offspring have methylation changes on the same site in a functional intronic region of the *FKBP5* gene (Yehuda et al., 2016). These effects were observed at bin 3/site 6 intron 7. According to these findings in two studies, it seems to be important to examine SNPs in intron 7 of *FKBP5* in the context of trauma effects.

We hypothesized that the influence of childhood trauma on brain volume might differ depending on the *FKBP5* genotype in non-clinical individuals. Gene and psychosocial environmental interaction effects might be influential to the brain regions. Such effects may play a critical role in predicting physiological and behavioral adaptations to stress including modulation of the HPA axis (Binder et al., 2008; Roy et al., 2010; Tatro et al., 2010; Zou et al., 2010; Supriyanto et al., 2011; Bevilacqua et al., 2012).

## Materials and Methods

### Participants

A total of 161 Korean non-clinical volunteers who lived in Seoul city and Gyeonggi province were initially included in the present study. They were recruited from the local community through flyers and posters. Participants with any history of neurological or other mental diseases were excluded from the study through the initial screening interviews, which were based on the Diagnostic and Statistical Manual of Mental Disorders, Fifth Edition. They were also excluded if they had shown any abnormal brain imaging findings. All participants had no history of psychiatric medical treatment and were not in need of any clinical mental health service/advice. Therefore, seven participants were excluded due to missing data from psychological variables; six additional participants were omitted from the analyses due to missing *FKBP5* gene data; and four participants who missed magnetic resonance imaging (MRI) and found abnormal MRI image were also excluded. A final sample of 144 non-clinical volunteers (44 men and 100 women) with a mean age of 46.93 ± 13.37 (years) was included. Study protocols were approved by the Institutional Review Board at Inje University Ilsan Paik Hospital (IRB no. 2015-07-025), and the study was conducted according to the Declaration of Helsinki. All participants provided a written informed consent prior to the study enrollment.

### Psychological Measures

#### Childhood Trauma Questionnaire

The Korean validated version of the Childhood Trauma Questionnaire (CTQ) was used to assess participants’ childhood trauma (Yu et al., 2009). The CTQ consists of five subscales of various childhood traumas, including emotional abuse, physical and sexual abuse, and emotional and physical neglect, and another scale for detecting minimization and denial. It is known as a useful self-report questionnaire in eliciting retrospective reports of childhood maltreatment from young adults (Everson et al., 2008). The CTQ consists of 28 items and is rated with a 5-point Likert scale ranging from 1 (“never true”) to 5 (“very often true”). Higher scores indicate more traumatic experiences in childhood. The coefficient alpha of the CTQ was 0.77 in the current study.

#### Hospital Anxiety and Depression Scale

The Korean-validated version of the Hospital Anxiety and Depression Scale (HADS) was used to assess anxiety and depression symptoms (Oh et al., 1999). It is a self-reported questionnaire with seven items for describing anxiety and with seven items for describing depression. It is assessed with a four-point Likert scale, ranging from 0 (no problems) to 3 (maximum distress). Higher scores indicate higher levels of anxiety and depression. The coefficients of each subtype of HADS were 0.87 (anxiety) and 0.78 (depression) in the current study.

#### Ruminative Response Scale

The Korean-validated version of the Ruminative Response Scale (RRS) was conducted to assess ruminative responses (Kim et al., 2010). It is comprised of three subscales specifically evaluating self-reproach, contemplation, and depressive rumination. It is a self-reported questionnaire with 22 items and is rated using a four-point Likert scale ranging from 1 (“almost never”) to 4 (“almost always”). Higher scores indicate higher levels of rumination responses. The coefficient alpha of the RRS was 0.94, and those of each subscale of the RRS were 0.86 (rumination), 0.89 (contemplation), and 0.91 (depressive rumination) in the current study.

#### World Health Organization Quality of Life Assessment Instrument

The Korean validated and abbreviated version of World Health Organization Quality of Life assessment instrument (WHOQOL) was used to assess the quality of life (Min et al., 2000). It consists of five subscales including physical health, psychological health, social relationships, environment, and general health. It is a self-reported questionnaire with 26 items and is rated using a 4-point Likert scale ranging from 1 (“almost never”) to 4 (“almost always”). Higher scores indicate higher levels of quality of life. The coefficient of the WHOQOL was 0.89 in the current study.

### Genetic Data

All participants had their blood sampled to extract DNA using a NanoDrop ND-1000 UV-Vis Spectrophotometer. Genomic DNA was then diluted to a 5-ng/μL concentration in 96-well polymerase chain reaction (PCR) plates. TaqMan SNP Genotyping Assays (Thermo Fisher Scientific, United States) were obtained, and the probes were labeled with FAM or VIC dye at the 5′ end and a minor-groove binder and non-fluorescent quencher at the 3′ end. PCR was done in 5 μL of a mixture containing 2 μL of a DNA sample, 0.125 μL of each TaqMan SNP Genotyping Assay (Thermo Fisher Scientific), 2.5 μL of TaqMan Genotyping Master Mix (Thermo Fisher Scientific), and 0.375 μL of distilled water. Amplification and detection were done with a detection system (QuantStudio 12K Flex Real-Time PCR System, Thermo Fisher Scientific) with the profile of 50°C for 2 min and 95°C for 10 min, followed by 60 cycles of 95°C for 15 s and 6°C for 1 min. After the PCR amplification, allelic discrimination was performed using the same machines (QuantStudio 12K Flex Real-Time PCR System), which was considered an endpoint plate read. The QuantStudio 12K Flex SOFTWARE calculated the fluorescence measurements made during the plate read and plotted Rn values based on the signals from each well. Finally, the analyzed plates were used to perform automatic or manual allele calls.

There were three positive samples and one negative control sample for each plate, and we confirmed positive controls with a clustering image. Our intra-genomic DNA samples of known genotypes were used for positive control. Ten *FKBP5* SNPs (rs9296158, rs3800373, rs1360780, rs9470080, rs4713916, rs4713919, rs6902321, rs56311918, rs3798345, and rs9380528) were genotyped to calculate ancestral proportions for all study participants. We calculated genotype frequencies for each individual polymorphism and evaluated the Hardy–Weinberg equilibrium to check the data quality and genotype error. The chi-squared test was used to compare the observed numbers of each genotype with those expected for the population following chi-squares distribution with one degree of freedom (Weir, 1996). All statistical tests and visualization of differentially expressed genes were conducted using R version 3.3.3.

In line with our study hypothesis and previous studies (Binder et al., 2008; Yun et al., 2020), all participants were divided into two genotype groups by allele frequencies (Table 1).

**TABLE 1:**
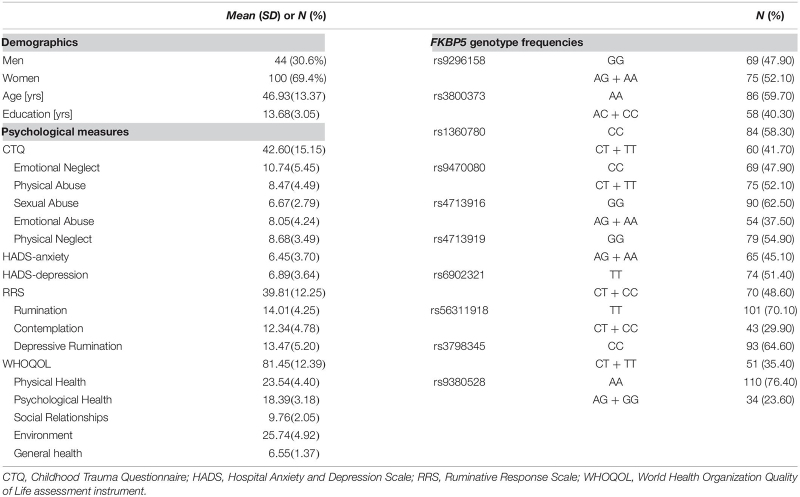
Demographics, psychological measures, and *FKBP5* variants of participants (*N* = 144).

### MRI Acquisition

Prior to MRI scanning, participants passed an MRI safety check and obtained a detailed explanation of the imaging protocol. Participants underwent MRI examination on a 1.5-T Magnetom Avanto (Siemens, Erlangen, Germany). To minimize the head motion, the manufacturer placed foam pads against each side of the head. A high-resolution T1-weighted MRI volume dataset was acquired (227 × 384 acquisition matrix, a 210 × 250 field-of-view, 0.9 × 0.7 × 1.2 voxel size, a total of 87,168 voxels, an echo time (TE) of 3.42 ms, a repetition time (TR) of 1,900 ms, slice thickness of 1.2 mm, and a flip angle of 15°).

The voxel-based volumetric analysis was conducted using computational Anatomy Toolbox 12 (developed by Christian Gaser, University of Jena)^1^ with the Statistical Parametric Mapping (SPM) 12 software package (Wellcome Department of Cognitive Neurology, London, United Kingdom) (Ashburner and Friston, 2005; Ashburner, 2007). Given the differences in the morphology of East Asian and Caucasian brains, the structural T1 images were registered to an ICBM East Asian template. Spatial normalization of the images was generated using the DARTEL algorithm (Ashburner, 2007). The images were then segmented into gray matter, white matter, and cerebrospinal fluid (Ashburner and Friston, 2005). Jacobian-transformed tissue probability maps were used to correct gray matter segments for volume differences. The volume of the regions was extracted using the Neuromorphometrics atlas, available in SPM 12, provided by Neuromorphometrics, Inc^2^.

### Regions of Interest

Fourteen regions of interest (i.e., seven regions of both hemispheres) were as follows: Several MRI studies have shown the interaction between *FKBP5* SNPs and the childhood trauma and how the structural and functional alterations of the hippocampus and amygdala (White et al., 2012; Pagliaccio et al., 2014; Holz et al., 2015) heavily relate with it. Likewise, volume alterations in the insula, superior and middle temporal gyrus, and anterior cingulate cortex were identified from abused individuals with the TT genotype of the *FKBP5* rs1360780 (Grabe et al., 2016). Other studies reported regions associated with both childhood trauma and *FKBP5* variants were amygdala, hippocampus, and OFC (Hart and Rubia, 2012; Fani et al., 2013; Pagliaccio et al., 2014; Holz et al., 2015; Han et al., 2017).

### Statistical Analysis

The genotypic distributions of the 10 SNPs located in *FKBP5* were evaluated by the Hardy–Weinberg Equilibrium. Normality for all psychological variables was tested using the skewness and kurtosis. Skewness less than 2 and kurtosis less than 7 were considered to be moderately normally distributed (Curran et al., 1996). All variables in our results were within the range of normal distribution. After checking for normality, Pearson’s correlation analysis with bootstrapping at a 5,000-sampling rate was performed. Following the correlation analysis, a regression analysis using Macro PROCESS for SPSS (version 2.16.3) was performed to examine the moderation effect of *FKBP5* variants on the relationship between childhood trauma and brain volume. CTQ was considered as a predictor, *FKBP5* variants were considered as moderators, and outcomes were considered as volume of brain regions. Age, sex, years of education, and total intracranial volume (TIV) were controlled as covariates. Anxiety and depression scale were also controlled, since a few participants who were enrolled had high scores in anxiety and depression scale. False discovery rate (FDR; Benjamini and Hochberg, 1995) with an adjusted *p* < 0.10 was used to correct for multiple comparisons. We used a significance level of 0.10 to alleviate excessive control.

Subsequently, the Johnson–Neyman technique (Hayes, 2013) was applied to probe an interaction effect. The Johnson–Neyman technique aligns the moderation variable in a continuous manner and computes the regions of significance for interactions by examining the significance between the predictor and outcome variables.

Finally, a partial correlation analysis was conducted on the brain regions where the moderation effect occurred to examine their relationship with psychological variables. The results were divided among each genotype of SNPs for comparison. Age, sex, years of education, TIV, anxiety, and depression scale were controlled as covariates. Five thousand bootstrapped samples were generated (Westfall, 2011). All statistical analyses were performed using SPSS version 21 (IBM Inc., Chicago, IL, United States).

## Results

### Descriptive Statistics

Demographics, psychological measures, and the genotype frequencies of the 10 *FKBP5* SNPs of participants are presented in Table 1. The genotypic distributions of the 10 SNPs located in *FKBP5* did not deviate from the Hardy–Weinberg Equilibrium (*p* > 0.05) (Zintzaras, 2010) except for rs9380528, which was excluded from the analysis. Demographics and psychological measures between genotypes of each SNP are shown in Supplementary Table 1. There were no significant differences in demographics and psychological measures between genotypes of *FKBP5* variants except for sex differences of rs1360780 and rs56311918.

### Moderation Effect

**FIGURE 1:**
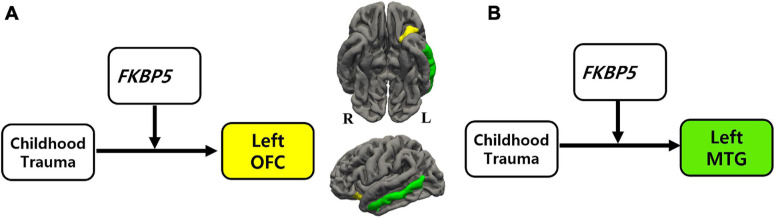
The moderation effect of *FKBP5* variants on the relationship between childhood trauma and brain volume. ^(a)^Survived six moderation effects of *FKBP5* variants (including rs3800373, rs1360780, rs4713916, rs4713919, rs6902321, and rs3798345) after FDR correction (alpha < 0.10) in the left OFC. ^(b)^Survived four moderation effects of *FKBP5* variants (including rs3800373, rs1360780, rs4713916, and rs3798345) after FDR correction (alpha < 0.10) in the left MTG. *FKBP5*, FK506-binding protein 5; OFC, orbitofrontal cortex; MTG, middle temporal gyrus.

Moderation effects which were statistically significant after applying the FDR correction for multiple comparisons (*p* < 0.10) are shown in Table 2. Therefore, six significant moderation effects of *FKBP5* variants on the relationship between CTQ and the left OFC were identified (Figure 1 and Supplementary Table 2). More specifically, the moderation model was significant in *FKBP5* rs3800373 (*R*^2^ = 0.591, *p* < 0.001), rs1360780 (*R*^2^ = 0.584, *p* < 0.001), rs4713916 (*R*^2^ = 0.581, *p* < 0.001), rs4713919 (*R*^2^ = 0.582, *p* < 0.001), rs6902321 (*R*^2^ = 0.762, *p* < 0.001), and rs3798345 (*R*^2^ = 0.586, *p* < 0.001). The main effects of CTQ and interaction effects between CTQ and *FKBP5* variants were significant in these six moderation models.

**TABLE 2:**
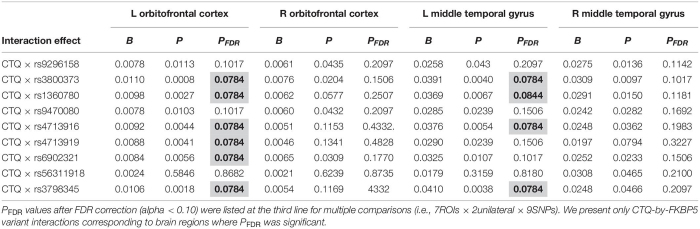
Statistically significant interaction effects of childhood trauma and *FKBP5* variants on alteration of brain volumes (*N* = 144).

Furthermore, four significant moderation effects of *FKBP5* variants on the relationship between CTQ and the left MTG were identified after FDR correction (Figure 1 and Supplementary Table 3). More specifically, the moderation model was significant in *FKBP5* rs3800373 (*R*^2^ = 0.697, *p* < 0.001), rs1360780 (*R*^2^ = 0.694, *p* < 0.001), rs4713916 (*R*^2^ = 0.692, *p* < 0.001), and rs3798345 (*R*^2^ = 0.696, *p* < 0.001). The main effects of CTQ and interaction effects between CTQ and *FKBP5* variants were significant in these four moderation models.

### Probing an Interaction

**FIGURE 2:**
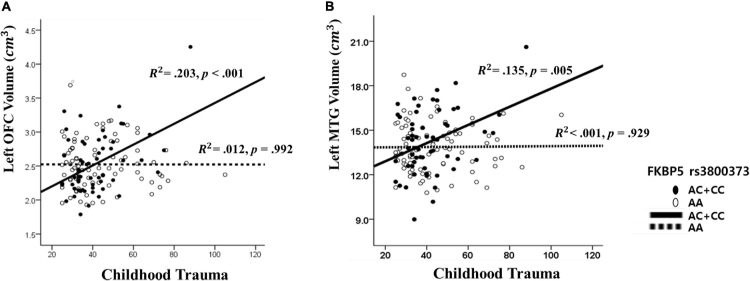
The interaction effect of *FKBP5* rs3800373 and childhood trauma on **(A)** the left orbitofrontal cortex and **(B)** the left Middle Temporal Gyrus. **(A)** The other interaction effects had similar patterns in rs1360780, rs4713916, rs4713919, rs6902321, and rs3798345. **(B)** The other interaction effects had similar patterns in rs1360780, rs4713916, and rs3798345.

The results from The Johnson–Neyman analysis indicated that in rs3800373, the positive relationship between CTQ and the left OFC volume was significant in those with AC + CC genotype (*B* = 0.011, *t* = 3.628, *p* < 0.001) (Figure 2). As for rs1360780, the positive relationship between CTQ and the left OFC volume was significant in those with the CT + TT genotype (*B* = 0.010, *t* = 3.628, *p* < 0.001). As for rs4713916, the positive relationship between CTQ and the left OFC volume was significant in those with the AG + AA genotype (*B* = 0.009, *t* = 3.494, *p* = 0.001). As for rs4713919, the positive relationship between CTQ and the left OFC volume was significant in those with the AG + AA genotype (*B* = 0.009, *t* = 3.539, *p* = 0.001). As for rs6902321, the positive relationship between CTQ and the left OFC volume was significant in those with the CT + CC genotype (*B* = 0.008, *t* = 3.450, *p* = 0.001). As for rs3798345, the positive relationship between CTQ and the left OFC volume was significant in those with CT + TT genotype (*B* = 0.001, *t* = 3.749, *p* = 0.001) (Supplementary Table 4).

Furthermore, as for rs3800373, the positive relationship between CTQ and the left MTG volume was significant in those with the AC + CC genotype (*B* = 0.042, *t* = 3.744, *p* < 0.001) (Figure 2). As for rs1360780, the positive relationship between CTQ and the left MTG volume was significant in those with the CT + TT genotype (*B* = 0.041, *t* = 3.628, *p* < 0.001). As for rs4713916, the positive relationship between CTQ and the left MTG volume was significant in those with the AG + AA genotype (*B* = 0.041, *t* = 3.666, *p* < 0.001). As for rs3798345, the positive relationship between CTQ and the left MTG volume was significant in those with the CT + TT genotype (*B* = 0.045, *t* = 3.728, *p* < 0.001) (Supplementary Table 4).

### Correlation Analysis

The left OFC was positively correlated with depressive rumination in those with the AC + CC genotype of rs3800373 (*r* = 0.314, *p* = 0.025), CT + TT genotype of rs1360780 (*r* = 0.320, *p* = 0.016), AG + AA genotype of rs4713916 (*r* = 0.366, *p* = 0.011), AG + AA genotype of rs4713919 (*r* = 0.259, *p* = 0.049), CT + CC genotype of rs6902321 (*r* = 0.307, *p* = 0.015), and CT + TT genotype of rs3798345 (*r* = 0.365, *p* = 0.015). Also, the left OFC was negatively correlated with physical health in those with the AC + CC genotype of rs3800373 (*r* = −0.298, *p* = 0.033), CT + TT genotype of rs1360780 (*r* = −0.278, *p* = 0.044), AG + AA genotype of rs4713916 (*r* = −0.351, *p* = 0.015), CT + CC genotype of rs6902321 (*r* = −0.264, *p* = 0.037), and CT + TT genotype of rs3798345 (*r* = −0.302, *p* = 0.047). Furthermore, the left OFC was negatively correlated with psychological health in those with the CT + CC genotype of rs6902321 (*r* = −0.309, *p* = 0.014) and CT + TT genotype of rs3798345 (*r* = −0.316, *p* = 0.037). In contrast, these correlations were not identified in those with the other genotype of *FKBP5* variants (Table 3).

**TABLE 3:**
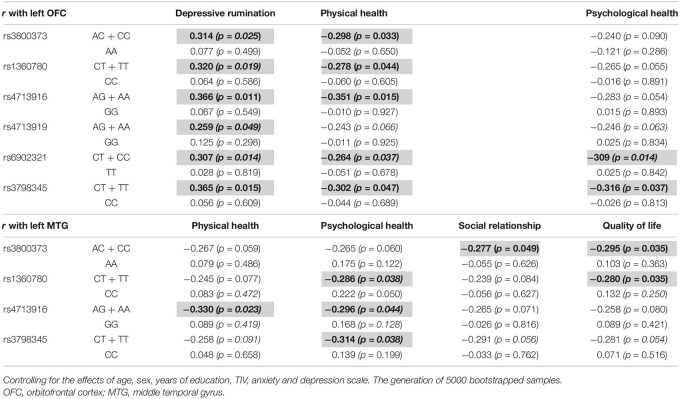
Brain regions affected by the interaction between *FKBP5* variants and the childhood trauma and the related psychological variables among genotypes.

The left MTG was negatively correlated with physical health in those with the AG + AA genotype of rs4713916 (*r* = −0.330, *p* = 0.023). The left MTG was negatively correlated with psychological health in those with the CT + TT genotype of rs1360780 (*r* = −0.286, *p* = 0.038), AG + AA genotype of rs4713916 (*r* = −0.296, *p* = 0.044), and CT + TT genotype of rs3798345 (*r* = −0.314, *p* = 0.038). The left MTG was negatively correlated with social relationship in those with the AC + CC genotype of rs3800373 (*r* = −0.277, *p* = 0.049). The left MTG was negatively correlated with quality of life in those with the AC + CC genotype of rs3800373 (*r* = −0.295, *p* = 0.035) and CT + TT genotype of rs1360780 (*r* = −0.280, *p* = 0.041). In contrast, these correlations were not identified in those with the other genotype of *FKBP5* variants (Table 3).

## Discussion

The present study aimed to explore the relationship among childhood trauma, 10 SNPs of *FKBP5*, and brain volume in non-clinical individuals. Our major findings were as follows. First, interaction of *FKBP5* variants and childhood trauma predicted volume alteration of the left OFC and left MTG. Second, individuals with the childhood trauma-susceptible genotype of *FKBP5* variants showed an enlarged volume of the left OFC and left MTG when childhood trauma was higher. Third, volume alteration of the left OFC and the left MTG showed significant negative correlations with positive feeling about life, and the left OFC showed significant positive correlations with negative cognition.

We found that the interaction of *FKBP5* and childhood trauma predicted volume alteration of the left OFC and left MTG in non-clinical individuals. A previous study reported that exposure to early life trauma has been shown to weaken developing brain structure and permanently set the stress response system on high alert (University CotDCaH, 2009). Furthermore, a genetic predisposition which is modulated or triggered by environmental factors could be a significant influencing factor on behavior and emotion (Saveanu and Nemeroff, 2012; Mandelli and Serretti, 2013). The *FKBP5* gene is an outstanding candidate that supports the model of stress-induced HPA-axis dysregulation in which genetically susceptible subjects are considered as long-standing biological risk (Grabe et al., 2016).

In our study, individuals with the childhood trauma-susceptible genotype of *FKBP5* variants showed enlarged volume of the left OFC when childhood trauma was higher. More specifically, the *FKBP5* genotype (“AC + CC” genotype of rs3800373, “CT + TT” genotype of rs1360780, “AG + AA” genotype of rs4713916, “AG + AA” genotype of rs4713919, CT + CC genotype of rs6902321, and “CT + TT” genotype of rs3798345) expressed an enlarged volume of the left OFC if they have shown higher scores of childhood trauma. Individuals with these six genotypes seemed to be susceptible to childhood trauma-related change of the left OFC according to a genetic vulnerability-stress theory (Caspi et al., 2003; Eley et al., 2004). In contrast, individuals with the other genotypes seemed to show no effect on brain volumetric change interacted by childhood trauma. Furthermore, enlarged volumes of the left MTG were found in the “AC + CC” genotype of rs3800373, “CT + TT” genotype of rs1360780, “AG + AA” genotype of rs4713916, and “CT + TT” genotype of rs3798345. Similarly, individuals with these three genotypes seemed to be susceptible to childhood trauma. In contrast, individuals with the other genotypes seemed to show no effect on brain volumetric change for childhood trauma.

Risk and protective genotype (or allele) of *FKBP5* are not well defined. For example, the rs3800373 “C” carriers appear to confer risk for stress-related disorders, whereas the “AA” genotype appears to be non-risk (Binder et al., 2008). In our study, the enlarged volumes of left OFC and left MTG were observed in those with the “C” carriers in the case of higher childhood traumatic experience. A specific genotype could not be conceptualized as neither inherently “good” nor “bad” (Halldorsdottir and Binder, 2017). It would be better to understand this genotype as having more responsivity (plasticity) or less responsivity (static) to environmental stress (Belsky et al., 2009). Our findings suggest that this susceptibility may also be determined by the severity of childhood traumatic experience.

Our findings suggested that enlarged volumes of the left OFC and the left MTG could imply neuroplastic compensatory mechanisms for protecting against environmental stress. In AC + CC genotypes, the volume of the left OFC showed significant positive correlations with negative cognition (e.g., depressive rumination) and negative correlations with positive feeling about life (e.g., physical health, psychological health). Previously, it was repeatedly reported that the OFC is important for adapting behavior about the emotional value of cues (Schoenbaum and Roesch, 2005; Roberts, 2006; Abler et al., 2009), cognitive evaluation of the environment, and expression of appropriate emotion-related responses (Rempel-Clower, 2007). OFC damage is associated with impaired control abilities (Hebscher et al., 2016) and neurotoxic effects in those related to pathological anxiety (de Wit et al., 2014). An extensive meta-analysis comprising 331 individuals with a history of childhood trauma and psychiatric comorbidities showed deficits in the OFC (Lim et al., 2014).

In susceptible-childhood trauma genotypes (i.e., “AC + CC” genotype of rs3800373, “CT + TT” genotype of rs1360780, “AG + AA” genotype of rs4713916, and “CT + TT” genotype of rs3798345), the volume of the left MTG was negatively correlated with positive feeling about life (e.g., physical health, psychological health, social relationship & quality of life). Previously, it was reported that the MTG is important for emotional memory processing, deductive reasoning, and recognition of emotional faces (Goel et al., 1998; Acheson and Hagoort, 2013; Meng et al., 2016). Thus, youth group who experienced physical abuse before the age of 12 (Lim et al., 2018) and adolescents with callous-unemotional conduct problems (De Brito et al., 2009) had greater middle temporal cortical volume than healthy controls. Yang et al. (2017) reported an enlarged volume of the left MTG linked to childhood traumas in first-episode major depressive disorder. The enlarged volume of the MTG in the non-chronic depression group and youth group can be a compensatory regulation for the early stage psychopathology of depression (Yang et al., 2017; Lim et al., 2018). In this context, our results showing enlarged volumes of the left OFC and the left MTG might be related to overworking as a function of compensatory cognitions (Brooks et al., 2016), which may prevent pathological anxiety and psychiatric symptoms.

Despite the significant findings reported, some limitations are present in this study. First, the current study is a cross-sectional design which presents a difficulty to disentangle whether alteration of the brain volume was present prior to the childhood trauma (McLaughlin et al., 2015). Second, we have a multiple testing issue for the correlation analysis between brain volumes and psychological variables.

In conclusion, this is one of the first studies to examine the brain structural effects of the gene–childhood trauma interaction with *FKBP5* variants in non-clinical individuals. Second, individuals with the childhood trauma-susceptible genotype of *FKBP5* variants showed enlarged volume of the left OFC and the left MTG when childhood trauma was higher. Our results suggested that enlarged volumes of the left OFC and the left MTG seem to imply a neuroplastic compensatory function of the brain protecting against environmental stresses.

## Data Availability Statement

The original contributions presented in the study are included in the article/Supplementary Material, further inquiries can be directed to the corresponding author.

## Ethics Statement

The studies involving human participants were reviewed and approved by IRB no. 2015-07-025. The patients/participants provided their written informed consent to participate in this study.

## Author Contributions

AK performed the methodology, formal analysis, project administration, and writing of the original draft. SK and HJ performed the material preparation, data collection, and software analysis. HL performed the visualization and english language editing. S-HL performed the funding acquisition, investigation, review, and supervision. All authors contributed to the study conception and design, discussed the results and commented on the manuscript at all stages, and approved the final manuscript.

## Conflict of Interest

The authors declare that the research was conducted in the absence of any commercial or financial relationships that could be construed as a potential conflict of interest.

## Footnotes

1 http://dbm.neuro.uni-jena.de/cat

2 http://neuromorphometrics.com
