# Supplementary material for: Influence of FKBP5 Variants and Childhood Trauma on Brain Volume in Non-clinical Individuals
Source: Front Behav Neurosci. 2021 Jun 3;15:663052. doi: 10.3389/fnbeh.2021.663052 (PMC8209293; doi:10.3389/fnbeh.2021.663052)
Supplement: Supplementary file 1 [file Table_1.DOCX]

**Supplementary Material**

**Influence of *FKBP5* variants and childhood trauma**

**on brain volume in non-clinical individuals**

**Aeran Kwon^1^, Sungkean Kim^2^, Hyeonjin Jeon^3^, Hyun Seo Lee^3^, Seung-Hwan Lee^3,4*^**

^1^Department of Social Welfare and Counseling, Chodang University, Muan, Republic of Korea

^2^Department of Human-Computer Interaction, Hanyang University, Ansan, Republic of Korea

^3^Clinical Emotion and Cognition Research Laboratory, Inje University, Goyang, Republic of Korea

^4^Department of Psychiatry, Inje University, Ilsan-Paik Hospital, Goyang, Republic of Korea

**Supplementary Table 1.** The demographics and psychological measures between genotypes of *FKBP5* variants

| **rs9296158** | **GG** | | **AG+AA** | | ***t* or *χ^2^*** | ***p*** |
| --- | --- | --- | --- | --- | --- | --- |
|  | ***Mean*(or *N*)** | ***SD*(or *%*)** | ***Mean*(or *N*)** | ***SD*(or *%*)** |  |  |
| **Demographics** |  |  |  |  |  |  |
| Men | 19 | 13 | 25 | 17 | .451 | .474 |
| Women | 50 | 35 | 50 | 35 |  |  |
| Age [yrs] | 47.90 | 12.78 | 46.04 | 13.91 | .832 | .407 |
| Education [yrs] | 13.70 | 3.04 | 13.67 | 3.09 | .057 | .955 |
| **Psychological measures** |  |  |  |  |  |  |
| CTQ | 44.71 | 17.01 | 40.67 | 13.02 | 1.591 | .114 |
| HADS-anxiety | 6.43 | 4.11 | 6.47 | 3.30 | -.052 | .959 |
| HADS-depression | 6.61 | 3.71 | 7.15 | 3.57 | -.886 | .377 |
| RRS | 39.07 | 13.10 | 40.49 | 11.46 | -.694 | .489 |
| Rumination | 13.81 | 4.40 | 14.19 | 4.13 | -.528 | .599 |
| Contemplation | 12.06 | 4.76 | 12.60 | 4.81 | -.679 | .498 |
| Depressive Rumination | 13.20 | 5.56 | 13.71 | 4.87 | -.579 | .563 |
| WHOQOL | 80.35 | 11.19 | 82.47 | 13.39 | -1.025 | .307 |
| Physical Health | 23.65 | 4.58 | 23.44 | 4.25 | .288 | .774 |
| Psychological Health | 18.06 | 2.84 | 18.70 | 3.45 | -1.214 | .227 |
| Social Relationships | 9.65 | 2.01 | 9.87 | 2.09 | -.625 | .533 |
| Environment | 25.36 | 4.33 | 26.08 | 5.41 | -.874 | .384 |
| general health | 6.52 | 1.27 | 6.57 | 1.46 | -.225 | .822 |

***Abbreviation***. CTQ, Childhood Trauma Questionnaire; HADS, Hospital Anxiety and Depression Scale; RRS, Ruminative Response Scale; WHOQOL, World Health Organization Quality of Life assessment instrument

| **rs3800373** | **AA** | | **AC+CC** | | ***t* or *χ^2^*** | ***p*** |
| --- | --- | --- | --- | --- | --- | --- |
|  | ***Mean*(or *N*)** | ***SD*(or *%*)** | ***Mean*(or *N*)** | ***SD*(or *%*)** |  |  |
| **Demographics** |  |  |  |  |  |  |
| Men | 21 | 15 | 23 | 16 | .020 | .065 |
| Women | 65 | 45 | 35 | 24 |  |  |
| Age [yrs] | 48.26 | 12.35 | 44.97 | 14.64 | 1.407 | .162 |
| Education [yrs] | 13.53 | 3.07 | 13.90 | 3.04 | -.696 | .488 |
| **Psychological measures** |  |  |  |  |  |  |
| CTQ | 43.50 | 16.49 | 41.28 | 12.94 | .905 | .367 |
| HADS-anxiety | 6.40 | 3.91 | 6.53 | 3.39 | -.221 | .826 |
| HADS-depression | 6.79 | 3.49 | 7.03 | 3.87 | -.393 | .695 |
| RRS | 39.72 | 12.91 | 39.95 | 11.30 | -.109 | .913 |
| Rumination | 13.97 | 4.38 | 14.07 | 4.09 | -.143 | .886 |
| Contemplation | 12.20 | 4.88 | 12.55 | 4.65 | -.435 | .664 |
| Depressive Rumination | 13.56 | 5.43 | 13.33 | 4.89 | .260 | .795 |
| WHOQOL | 80.55 | 10.55 | 82.79 | 14.70 | -1.003 | .319 |
| Physical Health | 23.52 | 4.35 | 23.57 | 4.50 | -.061 | .951 |
| Psychological Health | 18.02 | 2.77 | 18.95 | 3.66 | -1.622 | .108 |
| Social Relationships | 9.66 | 1.89 | 9.91 | 2.27 | -.719 | .473 |
| Environment | 25.38 | 4.24 | 26.26 | 5.79 | -.987 | .326 |
| general health | 6.44 | 1.29 | 6.71 | 1.48 | -1.141 | .256 |

***Abbreviation***. CTQ, Childhood Trauma Questionnaire; HADS, Hospital Anxiety and Depression Scale; RRS, Ruminative Response Scale; WHOQOL, World Health Organization Quality of Life assessment instrument

| **rs1360780** | **CC** | | **CT+TT** | | ***t* or** ***χ^2^*** | ***p*** |
| --- | --- | --- | --- | --- | --- | --- |
|  | ***Mean*(or *N*)** | ***SD*(or *%*)** | ***Mean*(or *N*)** | ***SD*(or *%*)** |  |  |
| **Demographics** |  |  |  |  |  |  |
| Men | 20 | 14 | 24 | 17 | .038 | .045 |
| Women | 64 | 44 | 36 | 25 |  |  |
| Age [yrs] | 48.07 | 12.43 | 45.33 | 14.54 | 1.182 | .239 |
| Education [yrs] | 13.48 | 3.07 | 13.97 | 3.04 | -.950 | .344 |
| **Psychological measures** |  |  |  |  |  |  |
| CTQ | 43.67 | 16.62 | 41.12 | 12.80 | 1.039 | .300 |
| HADS-anxiety | 6.44 | 3.90 | 6.47 | 3.42 | -.042 | .967 |
| HADS-depression | 6.76 | 3.52 | 7.07 | 3.81 | -.495 | .622 |
| RRS | 39.62 | 13.04 | 40.08 | 11.16 | -.223 | .823 |
| Rumination | 13.93 | 4.41 | 14.12 | 4.06 | -.261 | .794 |
| Contemplation | 12.23 | 4.94 | 12.50 | 4.58 | -.338 | .736 |
| Depressive Rumination | 13.46 | 5.45 | 13.47 | 4.87 | -.003 | .998 |
| WHOQOL | 80.49 | 10.61 | 82.80 | 14.51 | -1.050 | .296 |
| Physical Health | 23.56 | 4.40 | 23.52 | 4.43 | .057 | .954 |
| Psychological Health | 18.02 | 2.74 | 18.92 | 3.68 | -1.662 | .099 |
| Social Relationships | 9.65 | 1.91 | 9.92 | 2.24 | -.754 | .452 |
| Environment | 25.39 | 4.29 | 26.22 | 5.70 | -.945 | .347 |
| general health | 6.45 | 1.29 | 6.68 | 1.47 | -.999 | .320 |

***Abbreviation***. CTQ, Childhood Trauma Questionnaire; HADS, Hospital Anxiety and Depression Scale; RRS, Ruminative Response Scale; WHOQOL, World Health Organization Quality of Life assessment instrument

| **rs9470080** | **CC** | | **CT+TT** | | ***t* or *χ^2^*** | ***p*** |
| --- | --- | --- | --- | --- | --- | --- |
|  | ***Mean*(or *N*)** | ***SD*(or *%*)** | ***Mean*(or *N*)** | ***SD*(or *%*)** |  |  |
| **Demographics** |  |  |  |  |  |  |
| Men | 20 | 14 | 24 | 17 | .695 | .721 |
| Women | 49 | 34 | 51 | 39 |  |  |
| Age [yrs] | 47.33 | 13.13 | 46.56 | 13.66 | .346 | .730 |
| Education [yrs] | 13.88 | 3.03 | 13.49 | 3.08 | .766 | .445 |
| **Psychological measures** |  |  |  |  |  |  |
| CTQ | 44.13 | 16.74 | 41.20 | 13.49 | 1.161 | .248 |
| HADS-anxiety | 6.41 | 4.11 | 6.49 | 3.30 | -.141 | .888 |
| HADS-depression | 6.59 | 3.66 | 7.16 | 3.62 | -.932 | .353 |
| RRS | 39.22 | 13.10 | 40.36 | 11.47 | -.558 | .578 |
| Rumination | 13.77 | 4.39 | 14.23 | 4.13 | -.645 | .520 |
| Contemplation | 12.17 | 4.81 | 12.49 | 4.77 | -.400 | .690 |
| Depressive Rumination | 13.28 | 5.54 | 13.64 | 4.90 | -.419 | .676 |
| WHOQOL | 80.29 | 11.24 | 82.52 | 13.34 | -1.080 | .282 |
| Physical Health | 23.55 | 4.52 | 23.53 | 4.32 | .024 | .981 |
| Psychological Health | 18.14 | 2.86 | 18.62 | 3.46 | -.896 | .372 |
| Social Relationships | 9.61 | 2.04 | 9.91 | 2.07 | -.870 | .386 |
| Environment | 25.38 | 4.36 | 26.07 | 5.39 | -.840 | .403 |
| general health | 6.42 | 1.31 | 6.67 | 1.42 | -1.080 | .282 |

***Abbreviation***. CTQ, Childhood Trauma Questionnaire; HADS, Hospital Anxiety and Depression Scale; RRS, Ruminative Response Scale; WHOQOL, World Health Organization Quality of Life assessment instrument

| **rs4713916** | **GG** | | **AG+AA** | | ***t* or *χ^2^*** | ***p*** |
| --- | --- | --- | --- | --- | --- | --- |
|  | ***Mean*(or *N*)** | ***SD*(or *%*)** | ***Mean*(or *N*)** | ***SD*(or *%*)** |  |  |
| **Demographics** |  |  |  |  |  |  |
| Men | 23 | 16 | 21 | 15 | .093 | .098 |
| Women | 67 | 47 | 33 | 23 |  |  |
| Age [yrs] | 47.86 | 12.92 | 45.39 | 14.08 | 1.072 | .285 |
| Education [yrs] | 13.58 | 3.06 | 13.85 | 3.07 | -.520 | .604 |
| **Psychological measures** |  |  |  |  |  |  |
| CTQ | 43.21 | 16.06 | 41.59 | 13.57 | .619 | .537 |
| HADS-anxiety | 6.29 | 3.87 | 6.72 | 3.40 | -.680 | .498 |
| HADS-depression | 6.71 | 3.50 | 7.19 | 3.87 | -.756 | .451 |
| RRS | 39.56 | 12.86 | 40.24 | 11.26 | -.324 | .746 |
| Rumination | 13.82 | 4.42 | 14.31 | 3.97 | -.672 | .503 |
| Contemplation | 12.34 | 4.90 | 12.33 | 4.60 | .013 | .989 |
| Depressive Rumination | 13.39 | 5.39 | 13.59 | 4.91 | -.227 | .821 |
| WHOQOL | 80.89 | 10.77 | 82.39 | 14.77 | -.650 | .517 |
| Physical Health | 23.60 | 4.39 | 23.44 | 4.45 | .205 | .838 |
| Psychological Health | 18.21 | 2.84 | 18.70 | 3.69 | -.827 | .411 |
| Social Relationships | 9.77 | 1.97 | 9.76 | 2.20 | .021 | .983 |
| Environment | 25.47 | 4.32 | 26.19 | 5.80 | -.789 | .432 |
| general health | 6.44 | 1.29 | 6.72 | 1.48 | -1.181 | .240 |

***Abbreviation***. CTQ, Childhood Trauma Questionnaire; HADS, Hospital Anxiety and Depression Scale; RRS, Ruminative Response Scale; WHOQOL, World Health Organization Quality of Life assessment instrument

| **rs4713919** | **GG** | | **AG+AA** | | ***t* or *χ^2^*** | ***p*** |
| --- | --- | --- | --- | --- | --- | --- |
|  | ***Mean*(or *N*)** | ***SD*(or *%*)** | ***Mean*(or *N*)** | ***SD*(or *%*)** |  |  |
| **Demographics** |  |  |  |  |  |  |
| Men | 23 | 16 | 21 | 15 | .093 | .098 |
| Women | 67 | 47 | 33 | 23 |  |  |
| Age [yrs] | 47.86 | 12.92 | 45.39 | 14.08 | 1.072 | .285 |
| Education [yrs] | 13.58 | 3.06 | 13.85 | 3.07 | -.520 | .604 |
| **Psychological measures** |  |  |  |  |  |  |
| CTQ | 43.21 | 16.06 | 41.59 | 13.57 | .619 | .537 |
| HADS-anxiety | 6.29 | 3.87 | 6.72 | 3.40 | -.680 | .498 |
| HADS-depression | 6.71 | 3.50 | 7.19 | 3.87 | -.756 | .451 |
| RRS | 39.56 | 12.86 | 40.24 | 11.26 | -.324 | .746 |
| Rumination | 13.82 | 4.42 | 14.31 | 3.97 | -.672 | .503 |
| Contemplation | 12.34 | 4.90 | 12.33 | 4.60 | .013 | .989 |
| Depressive Rumination | 13.39 | 5.39 | 13.59 | 4.91 | -.227 | .821 |
| WHOQOL | 80.89 | 10.77 | 82.39 | 14.77 | -.650 | .517 |
| Physical Health | 23.60 | 4.39 | 23.44 | 4.45 | .205 | .838 |
| Psychological Health | 18.21 | 2.84 | 18.70 | 3.69 | -.827 | .411 |
| Social Relationships | 9.77 | 1.97 | 9.76 | 2.20 | .021 | .983 |
| Environment | 25.47 | 4.32 | 26.19 | 5.80 | -.789 | .432 |
| general health | 6.44 | 1.29 | 6.72 | 1.48 | -1.181 | .240 |

***Abbreviation***. CTQ, Childhood Trauma Questionnaire; HADS, Hospital Anxiety and Depression Scale; RRS, Ruminative Response Scale; WHOQOL, World Health Organization Quality of Life assessment instrument

| **rs6902321** | **TT** | | **CT+CC** | | ***t* or *χ^2^*** | ***p*** |
| --- | --- | --- | --- | --- | --- | --- |
|  | ***Mean*(or *N*)** | ***SD*(or *%*)** | ***Mean*(or *N*)** | ***SD*(or *%*)** |  |  |
| **Demographics** |  |  |  |  |  |  |
| Men | 22 | 15 | 22 | 15 | .825 | .858 |
| Women | 52 | 36 | 48 | 33 |  |  |
| Age [yrs] | 47.24 | 13.30 | 46.60 | 13.53 | .288 | .774 |
| Education [yrs] | 13.82 | 3.04 | 13.53 | 3.09 | .580 | .563 |
| **Psychological measures** |  |  |  |  |  |  |
| CTQ | 43.81 | 16.60 | 41.33 | 13.45 | .988 | .325 |
| HADS-anxiety | 6.38 | 4.02 | 6.53 | 3.35 | -.243 | .808 |
| HADS-depression | 6.55 | 3.64 | 7.24 | 3.63 | -1.137 | .257 |
| RRS | 39.58 | 12.92 | 40.06 | 11.59 | -.232 | .817 |
| Rumination | 13.88 | 4.38 | 14.14 | 4.14 | -.372 | .710 |
| Contemplation | 12.39 | 4.82 | 12.29 | 4.76 | .133 | .894 |
| Depressive Rumination | 13.31 | 5.49 | 13.63 | 4.91 | -.365 | .715 |
| WHOQOL | 80.89 | 11.46 | 82.04 | 13.36 | -.556 | .579 |
| Physical Health | 23.72 | 4.59 | 23.36 | 4.21 | .488 | .626 |
| Psychological Health | 18.24 | 2.94 | 18.55 | 3.43 | -.577 | .565 |
| Social Relationships | 9.69 | 2.03 | 9.84 | 2.09 | -.448 | .655 |
| Environment | 25.64 | 4.40 | 25.84 | 5.45 | -.252 | .801 |
| general health | 6.45 | 1.33 | 6.66 | 1.41 | -.925 | .356 |

***Abbreviation***. CTQ, Childhood Trauma Questionnaire; HADS, Hospital Anxiety and Depression Scale; RRS, Ruminative Response Scale; WHOQOL, World Health Organization Quality of Life assessment instrument

| **rs56311918** | **TT** | | **CT+CC** | | ***t* or *χ^2^*** | ***p*** |
| --- | --- | --- | --- | --- | --- | --- |
|  | ***Mean*(or *N*)** | ***SD*(or *%*)** | ***Mean*(or *N*)** | ***SD*(or *%*)** |  |  |
| **Demographics** |  |  |  |  |  |  |
| Men | 25 | 17 | 19 | 13 | .021 | .029 |
| Women | 76 | 53 | 24 | 17 |  |  |
| Age [yrs] | 47.75 | 12.84 | 45.00 | 14.51 | 1.132 | .260 |
| Education [yrs] | 13.57 | 3.10 | 13.93 | 2.97 | -.639 | .524 |
| **Psychological measures** |  |  |  |  |  |  |
| CTQ | 43.71 | 16.62 | 40.00 | 10.65 | 1.601 | .112 |
| HADS-anxiety | 6.37 | 3.91 | 6.65 | 3.17 | -.422 | .674 |
| HADS-depression | 6.79 | 3.60 | 7.12 | 3.75 | -.488 | .626 |
| RRS | 39.70 | 13.00 | 40.07 | 10.42 | -.164 | .870 |
| Rumination | 13.86 | 4.58 | 14.35 | 3.39 | -.708 | .481 |
| Contemplation | 12.40 | 4.86 | 12.21 | 4.63 | .214 | .831 |
| Depressive Rumination | 13.45 | 5.39 | 13.51 | 4.79 | -.070 | .945 |
| WHOQOL | 80.99 | 11.91 | 82.53 | 13.54 | -.683 | .495 |
| Physical Health | 23.70 | 4.58 | 23.16 | 3.96 | .673 | .502 |
| Psychological Health | 18.31 | 3.00 | 18.60 | 3.60 | -.493 | .623 |
| Social Relationships | 9.78 | 2.16 | 9.72 | 1.80 | .163 | .870 |
| Environment | 25.50 | 4.69 | 26.30 | 5.43 | -.900 | .369 |
| general health | 6.50 | 1.37 | 6.67 | 1.38 | -.719 | .473 |

***Abbreviation***. CTQ, Childhood Trauma Questionnaire; HADS, Hospital Anxiety and Depression Scale; RRS, Ruminative Response Scale; WHOQOL, World Health Organization Quality of Life assessment instrument

| **rs3798345** | **CC** | | **CT+TT** | | ***t* or *χ^2^*** | ***p*** |
| --- | --- | --- | --- | --- | --- | --- |
|  | ***Mean*(or *N*)** | ***SD*(or *%*)** | ***Mean*(or *N*)** | ***SD*(or *%*)** |  |  |
| **Demographics** |  |  |  |  |  |  |
| Men | 24 | 17 | 20 | 14 | .095 | .130 |
| Women | 69 | 48 | 31 | 22 |  |  |
| Age [yrs] | 47.47 | 13.00 | 45.94 | 14.09 | .656 | .513 |
| Education [yrs] | 13.47 | 3.03 | 14.06 | 3.09 | -1.102 | .272 |
| **Psychological measures** |  |  |  |  |  |  |
| CTQ | 43.18 | 16.27 | 41.55 | 12.95 | .660 | .511 |
| HADS-anxiety | 6.28 | 3.83 | 6.76 | 3.46 | -.752 | .453 |
| HADS-depression | 6.65 | 3.52 | 7.33 | 3.84 | -1.087 | .279 |
| RRS | 39.47 | 12.84 | 40.43 | 11.20 | -.448 | .655 |
| Rumination | 14.02 | 4.35 | 13.98 | 4.11 | .055 | .956 |
| Contemplation | 12.18 | 4.90 | 12.63 | 4.57 | -.533 | .595 |
| Depressive Rumination | 13.27 | 5.40 | 13.82 | 4.84 | -.611 | .542 |
| WHOQOL | 80.97 | 10.61 | 82.33 | 15.19 | -.570 | .570 |
| Physical Health | 23.68 | 4.38 | 23.29 | 4.46 | .499 | .619 |
| Psychological Health | 18.16 | 2.79 | 18.82 | 3.78 | -1.083 | .282 |
| Social Relationships | 9.76 | 1.95 | 9.76 | 2.24 | -.004 | .997 |
| Environment | 25.52 | 4.29 | 26.14 | 5.92 | -.660 | .511 |
| general health | 6.49 | 1.29 | 6.65 | 1.51 | -.638 | .524 |

***Abbreviation***. CTQ, Childhood Trauma Questionnaire; HADS, Hospital Anxiety and Depression Scale; RRS, Ruminative Response Scale; WHOQOL, World Health Organization Quality of Life assessment instrument

| **rs9380528** | **AA** | | **AG+GG** | | ***t* or *χ^2^*** | ***p*** |
| --- | --- | --- | --- | --- | --- | --- |
|  | ***Mean*(or *N*)** | ***SD*(or *%*)** | ***Mean*(or *N*)** | ***SD*(or *%*)** |  |  |
| **Demographics** |  |  |  |  |  |  |
| Men | 34 | 24 | 10 | 7 | .868 | 1.000 |
| Women | 76 | 53 | 24 | 17 |  |  |
| Age [yrs] | 46.11 | 13.47 | 49.59 | 12.88 | -1.330 | .186 |
| Education [yrs] | 13.67 | 3.03 | 13.71 | 3.18 | -.055 | .956 |
| **Psychological measures** |  |  |  |  |  |  |
| CTQ | 42.86 | 15.74 | 41.76 | 13.24 | .369 | .713 |
| HADS-anxiety | 6.73 | 3.69 | 5.56 | 3.62 | 1.620 | .107 |
| HADS-depression | 6.85 | 3.62 | 7.00 | 3.73 | -.203 | .839 |
| RRS | 40.00 | 12.30 | 39.21 | 12.24 | .329 | .742 |
| Rumination | 14.22 | 4.29 | 13.32 | 4.10 | 1.073 | .285 |
| Contemplation | 12.38 | 4.71 | 12.21 | 5.06 | .187 | .852 |
| Depressive Rumination | 13.40 | 5.34 | 13.68 | 4.78 | -.287 | .775 |
| WHOQOL | 81.27 | 12.03 | 82.03 | 13.66 | -.310 | .757 |
| Physical Health | 23.56 | 4.33 | 23.47 | 4.68 | .107 | .915 |
| Psychological Health | 18.31 | 3.22 | 18.65 | 3.06 | -.536 | .593 |
| Social Relationships | 9.86 | 1.88 | 9.44 | 2.54 | 1.050 | .296 |
| Environment | 25.56 | 4.70 | 26.29 | 5.62 | -.755 | .451 |
| general health | 6.48 | 1.33 | 6.76 | 1.50 | -1.054 | .294 |

***Abbreviation***. CTQ, Childhood Trauma Questionnaire; HADS, Hospital Anxiety and Depression Scale; RRS, Ruminative Response Scale; WHOQOL, World Health Organization Quality of Life assessment instrument

**Supplementary Table 2.** Regression analysis examining moderation effect of *FKBP5* variants in the relationship between childhood trauma and left OFC (*N*=144)

|  | **Predictor** | ***B*** | ***SE*** | ***t*** | ***p*** | ***R^2^*** |  |  | **Predictor** | ***B*** | ***SE*** | ***t*** | ***p*** | ***R^2^*** |
| --- | --- | --- | --- | --- | --- | --- | --- | --- | --- | --- | --- | --- | --- | --- |
| Left  OFC | (Constant) | 1.295 | .407 | 3.183 | .002 | .591*** |  | Left  OFC | (Constant) | 1.220 | .410 | 2.980 | .003 | .584*** |
|  | age | -.009 | .002 | -5.096 | .000 |  |  |  | age | -.009 | .002 | -5.116 | .000 |  |
|  | sex | -.022 | .055 | -.397 | .692 |  |  |  | sex | -.018 | .055 | -.319 | .750 |  |
|  | education | -.004 | .008 | -.497 | .620 |  |  |  | education | -.004 | .008 | -.511 | .610 |  |
|  | TIV | .002 | .000 | 9.234 | .000 |  |  |  | TIV | .002 | .000 | 9.188 | .000 |  |
|  | anxiety | -.010 | .008 | -1.256 | .211 |  |  |  | anxiety | -.010 | .008 | -1.317 | .190 |  |
|  | depression | .003 | .008 | .404 | .687 |  |  |  | depression | .004 | .008 | .498 | .619 |  |
|  | rs3800373 | -.468 | .141 | -3.317 | .**001** |  |  |  | rs1360780 | -.416 | .142 | -2.924 | **.004** |  |
|  | CTQ | -.011 | .004 | -2.463 | **.015** |  |  |  | CTQ | -.009 | .004 | -2.108 | **.037** |  |
|  | rs3800373  × CTQ | .011 | .003 | 3.441 | **.001** |  |  |  | rs1360780  × CTQ | .010 | .003 | 3.053 | **.003** |  |
|  |  |  |  |  |  |  |  |  |  |  |  |  |  |  |
| Left OFC | (Constant) | 1.120 | .397 | 2.819 | .006 | .581*** |  | Left  OFC | (Constant) | 1.127 | .392 | 2.872 | .005 | .582*** |
|  | age | -.009 | .002 | -5.421 | .000 |  |  |  | age | -.010 | .002 | -5.550 | .000 |  |
|  | sex | -.007 | .055 | -.122 | .903 |  |  |  | sex | .003 | .055 | .055 | .956 |  |
|  | education | -.003 | .008 | -.413 | .680 |  |  |  | education | -.005 | .008 | -.573 | .568 |  |
|  | TIV | .002 | .000 | 9.450 | .000 |  |  |  | TIV | .002 | .000 | 9.571 | .000 |  |
|  | anxiety | -.010 | .008 | -1.330 | .186 |  |  |  | anxiety | -.010 | .008 | -1.253 | .212 |  |
|  | depression | .005 | .008 | .538 | .591 |  |  |  | depression | .005 | .008 | .619 | .537 |  |
|  | rs4713916 | -.385 | .141 | -2.723 | .**007** |  |  |  | rs4713919 | -.379 | .133 | -2.855 | **.005** |  |
|  | CTQ | -.009 | .004 | -1.950 | .053 |  |  |  | CTQ | -.009 | .004 | -1.987 | **.049** |  |
|  | rs4713916  × CTQ | .009 | .003 | 2.896 | **.004** |  |  |  | rs4713919  × CTQ | .009 | .003 | 2.922 | **.004** |  |
|  |  |  |  |  |  |  |  |  |  |  |  |  |  |  |
| Left  OFC | (Constant) | 1.171 | .402 | 2.918 | .004 | .584*** |  | Left  OFC | (Constant) | 1.213 | .403 | 3.007 | .003 | .586*** |
|  | age | -.009 | .002 | -5.437 | .000 |  |  |  | age | -.009 | .002 | -5.301 | .000 |  |
|  | sex | -.010 | .055 | -.185 | .854 |  |  |  | sex | -.008 | .055 | -.138 | .890 |  |
|  | education | -.004 | .008 | -.508 | .613 |  |  |  | education | -.003 | .008 | -.370 | .712 |  |
|  | TIV | .002 | .000 | 9.388 | .000 |  |  |  | TIV | .002 | .000 | 9.183 | .000 |  |
|  | anxiety | -.010 | .008 | -1.234 | .219 |  |  |  | anxiety | -.010 | .008 | -1.290 | .200 |  |
|  | depression | .004 | .008 | .528 | .599 |  |  |  | depression | .004 | .008 | .471 | .639 |  |
|  | rs6902321 | -.377 | .133 | -2.830 | **.005** |  |  |  | rs3798345 | -.441 | .148 | -2.983 | **.003** |  |
|  | CTQ | -.008 | .004 | -1.906 | .059 |  |  |  | CTQ | -.010 | .004 | -2.224 | **.028** |  |
|  | rs6902321  × CTQ | .008 | .003 | 2.817 | **.006** |  |  |  | rs3798345  × CTQ | .011 | .003 | 3.177 | **.002** |  |
|  |  |  |  |  |  |  |  |  |  |  |  |  |  |  |

*** *p*<.001; ***Note.*** Each of these are statistically significant at the *alpha* < .10 level after false discovery rate correction for multiple comparisons (i.e., 7ROIs × 2unilateral × 9SNPs). ***Abbreviation***. CTQ, Childhood trauma questionnaire; *FKBP5*, FK506-binding protein 5; OFC, orbitofrontal cortex

**Supplementary Table 3.** Regression analysis examining moderation effect of *FKBP5* variants in the relationship between childhood trauma and left MTG (*N*=144).

|  | **Predictor** | ***B*** | | ***SE*** | | ***t*** | | ***p*** | | ***R^2^*** |  |  | **Predictor** | | ***B*** | | ***SE*** | | ***t*** | | ***p*** | | ***R^2^*** |
| --- | --- | --- | --- | --- | --- | --- | --- | --- | --- | --- | --- | --- | --- | --- | --- | --- | --- | --- | --- | --- | --- | --- | --- |
| Left  MTG | (Constant) | | 3.666 | | 1.705 | | 2.150 | | .033 | .697*** |  | Left  MTG | | (Constant) | 3.647 | 1.710 | | 2.133 | | .035 | | .694*** | |
|  | age | | -.022 | | .007 | | -3.048 | | .003 |  |  |  |  | age | -.023 | .007 | | -3.122 | | .002 | |  |  |
|  | sex | | -.229 | | .230 | | -.998 | | .320 |  |  |  |  | sex | -.224 | .231 | | -.970 | | .334 | |  |  |
|  | education | | -.015 | | .033 | | -.470 | | .639 |  |  |  |  | education | -.017 | .033 | | -.515 | | .608 | |  |  |
|  | TIV | | .009 | | .001 | | 12.811 | | .000 |  |  |  |  | TIV | .009 | .001 | | 12.730 | | .000 | |  |  |
|  | anxiety | | .043 | | .032 | | 1.332 | | .185 |  |  |  |  | anxiety | .042 | .032 | | 1.299 | | .196 | |  |  |
|  | depression | | -.036 | | .034 | | -1.059 | | .292 |  |  |  |  | depression | -.035 | .035 | | -1.009 | | .315 | |  |  |
|  | rs3800373 | | -1.307 | | .591 | | -2.211 | | **.029** |  |  |  |  | rs1360780 | -1.240 | .594 | | -2.090 | | **.039** | |  |  |
|  | CTQ | | -.036 | | .018 | | -1.964 | | .052 |  |  |  |  | CTQ | -.033 | .018 | | -1.809 | | .073 | |  |  |
|  | rs3800373  × CTQ | | .039 | | .013 | | 2.926 | | **.004** |  |  |  |  | rs1360780  × CTQ | .037 | .013 | | 2.754 | | **.007** | |  |  |
|  |  |  |  | |  | |  | |  |  |  |  | |  |  |  | |  | |  | |  | |
| Left MTG | (Constant) | | 3.792 | | 1.658 | | 2.287 | | .024 | 692*** |  | Left MTG | | (Constant) | 3.854 | 1.681 | | 2.293 | | .023 | | . 696*** | |
|  | age | | -.025 | | .007 | | -3.449 | | .001 |  |  |  |  | age | -.024 | .007 | | -3.367 | | .001 | |  |  |
|  | sex | | -.219 | | .229 | | -.955 | | .341 |  |  |  |  | sex | -.205 | .228 | | -.899 | | .370 | |  |  |
|  | education | | -.011 | | .033 | | -.345 | | .731 |  |  |  |  | education | -.014 | .033 | | -.425 | | .672 | |  |  |
|  | TIV | | .009 | | .001 | | 12.867 | | .000 |  |  |  |  | TIV | .009 | .001 | | 12.731 | | .000 | |  |  |
|  | anxiety | | .039 | | .033 | | 1.190 | | .236 |  |  |  |  | anxiety | .041 | .032 | | 1.264 | | .209 | |  |  |
|  | depression | | -.031 | | .035 | | -.907 | | .366 |  |  |  |  | depression | -.036 | .034 | | -1.037 | | .301 | |  |  |
|  | rs4713916 | | -1.352 | | .590 | | -2.294 | | **.023** |  |  |  |  | rs3798345 | -1.405 | .617 | | -2.278 | | **.024** | |  |  |
|  | CTQ | | -.035 | | .018 | | -1.904 | | .059 |  |  |  |  | CTQ | -.037 | .018 | | -2.011 | | **.046** | |  |  |
|  | rs4713916  × CTQ | | .038 | | .013 | | 2.830 | | **.005** |  |  |  | | rs3798345  × CTQ | .041 | .014 | | 2.948 | | **.004** | |  |  |

*** *p*<.001; ***Note.*** Each of these are statistically significant at the *alpha* < .10 level after false discovery rate correction for multiple comparisons (i.e., 7ROIs × 2unilateral × 9SNPs). ***Abbreviation***. CTQ, Childhood trauma questionnaire; *FKBP5*, FK506-binding protein 5; MTG, middle temporal gyrus

**Supplementary Table 4.** The effect of the childhood trauma on volume alteration of the left orbitofrontal cortex between *FKBP5* genotype in non-clinical individuals (*N*=144)

|  | ***FKBP5*** | **Genotype** | **Effect** | ***SE*** | ***t*** | ***p*** | **LLCI** | **ULCI** |
| --- | --- | --- | --- | --- | --- | --- | --- | --- |
| **CTQ 🡪 left OFC** | **rs3800373** | **AA** | .000 | .002 | .196 | .845 | -.003 | .004 |
|  |  | **AC+CC** | .011 | .003 | 3.990 | <.001 | .006 | .017 |
|  |  |  |  |  |  |  |  |  |
|  | **rs1360780** | **CC** | .000 | .002 | .245 | .807 | -.003 | .004 |
|  |  | **CT+TT** | .010 | .003 | 3.628 | <.001 | .005 | .015 |
|  |  |  |  |  |  |  |  |  |
|  | **rs4713916** | **GG** | .001 | .002 | .342 | .733 | -.003 | .004 |
|  |  | **AG+AA** | .009 | .003 | 3.494 | .001 | .004 | .015 |
|  |  |  |  |  |  |  |  |  |
|  | **rs4713919** | **GG** | .000 | .002 | .043 | .966 | -.004 | .004 |
|  |  | **AG+AA** | .009 | .002 | 3.539 | .001 | .004 | .014 |
|  |  |  |  |  |  |  |  |  |
|  | **rs6902321** | **TT** | .000 | .002 | -.005 | .996 | -.004 | .004 |
|  |  | **CT+CC** | .008 | .002 | 3.450 | .001 | .004 | 0.013 |
|  |  |  |  |  |  |  |  |  |
|  | **rs3798345** | **CC** | .001 | .002 | .377 | .707 | -.003 | .004 |
|  |  | **CT+TT** | .011 | .003 | 3.749 | <.001 | .005 | .017 |

|  | | ***FKBP5*** | | **Genotype** | | **Effect** | | ***SE*** | | ***t*** | | ***p*** | | **LLCI** | | **ULCI** | |
| --- | --- | --- | --- | --- | --- | --- | --- | --- | --- | --- | --- | --- | --- | --- | --- | --- | --- |
|  | **rs3800373** | | **AA** | | .003 | | .007 | | .409 | | .683 | | -.011 | | .017 | |  |
| **CTQ 🡪 left MTG** |  | | **AC+CC** | | .042 | | .011 | | 3.744 | | <.001 | | .020 | | .065 | |  |
|  |  | |  | |  | |  | |  | |  | |  | |  | |  |
|  | **rs1360780** | | **CC** | | .003 | | .007 | | .441 | | .660 | | -.011 | | .017 | |  |
|  |  | | **CT+TT** | | .041 | | .011 | | 3.628 | | <.001 | | .019 | | .063 | |  |
|  |  | |  | |  | |  | |  | |  | |  | |  | |  |
|  | **rs4713916** | | **GG** | | .002 | | .007 | | .334 | | 0.739 | | -.012 | | .017 | |  |
|  |  | | **AG+AA** | | .041 | | .011 | | 3.666 | | <.001 | | .019 | | .063 | |  |
|  |  | |  | |  | |  | |  | |  | |  | |  | |  |
|  | **rs3798345** | | **CC** | | .003 | | .007 | | .493 | | 0.623 | | -.010 | | .017 | |  |
|  |  | | **CT+TT** | | .045 | | .012 | | 3.728 | | <.001 | | .021 | | .069 | |  |
